# Effect of dexamethasone on hypothalamic expression of appetite-related genes in chickens under different diet and feeding conditions

**DOI:** 10.1186/s40104-016-0084-x

**Published:** 2016-04-12

**Authors:** Lei Liu, Shaohua Xu, Xiaojuan Wang, Hongchao Jiao, Jingpeng Zhao, Hai Lin

**Affiliations:** Department of Animal Science, Shandong Agricultural University, Shandong Key Lab for Animal Biotechnology and Disease Control, Taian, Shandong 271018 China

**Keywords:** Chickens, Glucocorticoids, Hypothalamus, Neuropeptides

## Abstract

**Background:**

Glucocorticoids (GCs) are involved in the control of appetite in birds and mammals. The effect of GCs on feed intake in birds depends on their dietary energy level. But the regulation mechanism of GCs on appetite is still unclear in chickens facing to different energy level. An experiment was conducted to investigate the effect of dexamethasone (DEX) on hypothalamic expression of appetite-related peptides in chickens fed high/low fat diet and under fasting/feeding condition.

**Results:**

An interaction between DEX injection and dietary energy level was found on hypothalamic corticotropin-releasing hormone (CRH) gene expression in fasted chickens (*P* < 0.05). The chickens, given a DEX injection and a low fat diet treatment, had the highest CRH mRNA levels than any of the fasted chickens given treatments (*P* < 0.05). Under fasting conditions, the DEX treatment significantly increased hypothalamic neuropeptide Y (NPY) and GC receptors mRNA levels (*P* < 0.05). Under re-feeding conditions, DEX treatment significantly decreased hypothalamic expression levels of NPY and agouti-related peptide (AgRP) but significantly increased the level of hypothalamic CRH expression (*P* < 0.05).

**Conclusion:**

A regulatory network formed by NPY, AgRP and CRH is associated with the appetite-control by GCs. The result suggests that the regulation of GCs on orexigenic neuropeptides expression is dependent at least partially on dietary energy level and feeding state.

**Electronic supplementary material:**

The online version of this article (doi:10.1186/s40104-016-0084-x) contains supplementary material, which is available to authorized users.

## Background

Hypothalami play a pivotal role in influencing feed intake in mammals and birds [1]. There are a population of neurons influencing appetite in hypothalamus, such as orexigenic neuropeptides (e.g., neuropeptide Y [NPY], agouti-related peptide [AgRP]) [1], and anorexigenic neuropeptides (e.g., proopiomelanocortin [POMC], and corticotropin-releasing hormone, [CRH]) [2, 3]. The release of these neuropeptides is closely associated with the feeding state and dietary energy level. For example, the fasting for 24 h decreased the hypothalamic POMC and CRH genes expression, but increased the AgRP gene expression in chicks [4]. The mice fed with high fat diet had the lower NPY and AgRP mRNA levels [5, 6].

Peripheral signal (e.g., insulin and glucocorticoids [GCs]) is integrated in hypothalamic arcuate nucleus (ARC) of mammals or infundibular nucleus of birds [7, 8]. GCs are involved in the appetite-control. Intracerebroventricular (ICV) injection of GCs increases feed intake in rats and chicks [8, 9]. Additionally, the effect of GCs on feed intake in birds depends on its dosage. Feed intake increased in chickens that were given high dosages of GCs [10]. Long-term peripheral corticosterone administration increased feed consumption on the comparable body weight basis [11, 12]. Bartov [13] showed that the effect of GCs on appetite was diet-type dependent, and GCs could stimulate feed intake of high-protein diet. Besides, fasting could not only alter the hypothalamic appetite-related genes expression [14], but also increase the blood corticosterone level [15]. GC receptors (GR) are located in hypothalamus and essential for energy balance regulation [16]. GCs stimulate NPY expression, while restraining POMC synthesis and release in birds and mammals via GR [17, 18]. Therefore, we hypothesized that peripheral GC-induced gene expression of appetitive neuropeptides could be changed by dietary energy level as well as the feeding state.

DEX, a synthetic glucocorticoid exhibiting a high affinity for GR and a delayed plasma clearance [19], was employed in the present study to induce the hyper GCs status. And we examined the effect of GCs on hypothalamic appetitive peptides in chickens fed high/low fat diet and under fasting/feeding condition and determined the major appetitive peptides that respond to peripheral GCs.

## Methods

### Animals

Male broiler (Arbor Acres) chicks of 1 d were reared in an environmentally controlled room. Temperature and lighting was maintained in accordance with commercial conditions. The composition and nutrient levels of the diets of the chickens used in experiment 1 are listed in Additional file 1: Table S1. All animal experiments were reviewed and approved by the Institutional Animal Care and Use Committee of Shandong Agricultural University and performed in accordance with the “Guidelines for Experimental Animals” of the Ministry of Science and Technology (Beijing, PR China). Animal suffering was minimized as much as possible.

### Experimental protocol and sample collection

At 1 d of age, 64 chickens of similar body weight were divided into two groups, with four replicates per group and eight chickens per replicate. Chickens were randomly subjected to one of the following two treatments: 1) feeding with high-fat diet (HFD, 15.06 MJ/kg, 13.5 % soy oil), or 2) feeding with low-fat diet (LFD, 10.90 MJ/kg, 0 % soy oil). At 35 d of age, every replicate was either fasted for 24 h and given a dexamethasone injection (DEX, 1 mg/kg body weight/time, subcutaneous injection at 0 and 12 h after fasting), or fasted for 24 h plus a saline injection (Control, same volume as the DEX group, subcutaneous injection 0 and 12 h after fasting) [20, 21]. After a 24-h period of fasting, all groups were re-fed for 3 h with the same diet at 40 g/bird that they received before the fasting period. At the end of the fasting and re-feeding period, two chickens from each replicate were selected and sacrificed. A blood sample was drawn from a wing vein using a heparinised syringe. Plasma was obtained following centrifugation at 400 g for 10 min at 4 °C and then stored at −20 °C. Hypothalami were collected according to Yuan et al. [22]. After being flash-frozen in liquid nitrogen, the hypothalami were stored at −80 °C for subsequent RNA extraction.

### Measurement of plasma insulin level

Plasma glucose concentration was measured spectrophotometrically with commercial diagnostic kits (Hitachi High-Technologies Corp.; Jiancheng Bioengineering Institute, Nanjing, P.R. China). Plasma insulin was measured using radioimmunoassay with guinea pig anti-porcine insulin serum (3 V Bio-engineering group Co., Weifang, P.R. China). Which have been successfully applied in poultry research [13].

### RNA isolation and analysis

Total RNA extraction and qRT-PCR were performed as described previously [7]. Sequences of primers are shown in Table 1. The PCR data were analyzed with the 2^−ΔΔCT^ method [23]. The mRNA levels of target genes were normalised to glyceraldehyde 3-phosphate dehydrogenase (GAPDH) mRNA and 18S ribosomal RNA (18SrRNA) (ΔCT). On the basis of the Ct values, 18S or GAPDH mRNA expression was stable across the treatments in this study (*P* > 0.1). The group with only LFD treatment was deemed to be 1.

**Table 1:** Gene-specific primers used for the analysis of chicken gene expression

Gene	GenBank accession no.	Primer sequences (5′-3′)	Product size, bp
GAPDH	NM_204305	F: ACATGGCATCCAAGGAGTGAG	266
		R: GGGGAGACAGAAGGGAACAGA	
18S	AF173612	F: ATAACGAACGAGACTCTGGCA	136
		R: CGGACATCTAAGGGCATCACA	
NPY	M87294	F: GAGGCACTACATCAACCTCATCAC	101
		R: TGTTTTCTGTGCTTTCCCTCAA	
CRH	NM_001123031	F: CTCCCTGGACCTGACTTTCC	86
		R: TGTTGCTGTGGGCTTGCT	
AgRP	NM_001031457	F: GGAACCGCAGGCATTGTC	163
		R: GTAGCAGAAGGCGTTGAAGAA	
POMC	NM_001031098	F: CGCTACGGCGGCTTCA	88
		R: TCTTGTAGGCGCTTTTGACGAT	
GR	DQ227738	F: CATGAACCTCGAAGCTCGCAAGA	159
		R: ACCTCCAGCAGTGACACCAG	

### Statistical analysis

The data are presented as the mean ± SEM. A two-way ANOVA model was used to analyze the primary effects of GCs and the dietary energy treatments as well as their interactions, using Statistical Analysis Systems statistical software package (Version 8e, SAS Institute, Cary, NC, USA). When the main effect of the treatment was significant in the analysis, the differences between means were assessed by Duncan’s multiple range analysis. The mean was considered significantly different at *P* < 0.05.

## Results

**Fig. 1:**
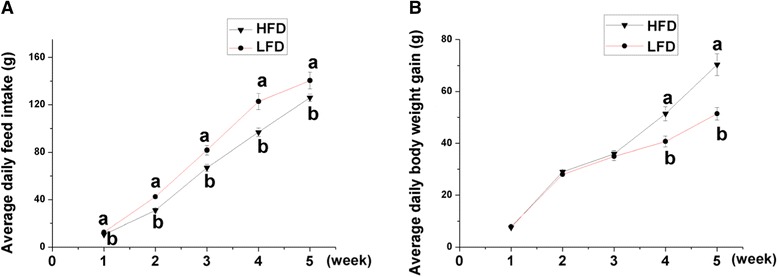
Effects of Dietary fat levels on average daily feed intake (**a**) and body weight gain (**b**). Values indicate mean ± SEM (*n* = 8). ^a, b^ Means with different superscripts are significantly different (*P* < 0.05)

The food intake in LFD-fed chickens was significantly higher than that of the HFD-fed ones in whole period (*P* < 0.05, Fig. 1a), but the body weight gain revealed the opposite tendency from the fourth week (*P* < 0.05, Fig. 1b).

**Fig. 2:**
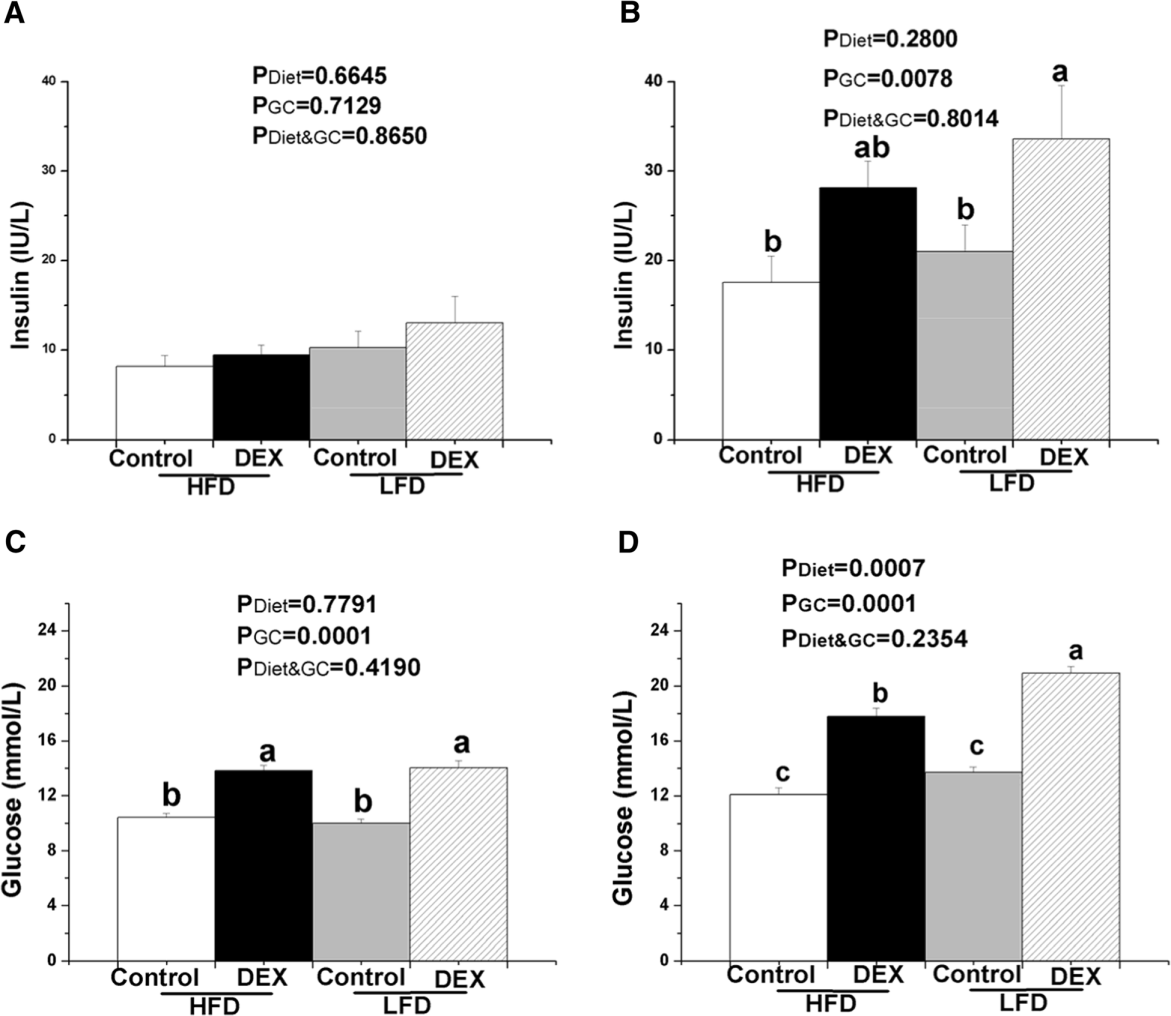
Effects of GCs on plasma insulin level in chickens. **a**  and **c** chickens under fasting for 24 h condition, **b** and **d** chickens under re-feeding for 3 h after fasting condition. Values indicate mean ± SEM (*n* = 8). ^a, b^ Means with different superscripts are significantly different (*P* < 0.05)

Dietary fat did not affect the plasma insulin concentration in fasted and re-fed chickens (*P* > 0.05, Fig. 2a). However, DEX injection significantly increased the plasma insulin concentration in re-fed chickens (*P* < 0.05, Fig. 2 b). In fasting chickens, DEX treatment significantly increased the plasma glucose concentration (*P* < 0.05), but diet treatment had no significant effect (*P* > 0.05, Fig. 2c). Both diet and DEX treatments had an effect on the plasma glucose concentration in re-fed chickens, and LFD and DEX treatments significantly increased the plasma glucose concentration (*P* < 0.05, Fig. 2d). As shown in Fig. 2, no effect of the interaction between DEX injection and dietary fat on plasma insulin and glucose concentration was detected in fasted and re-fed chickens (*P* > 0.05).

**Fig. 3:**
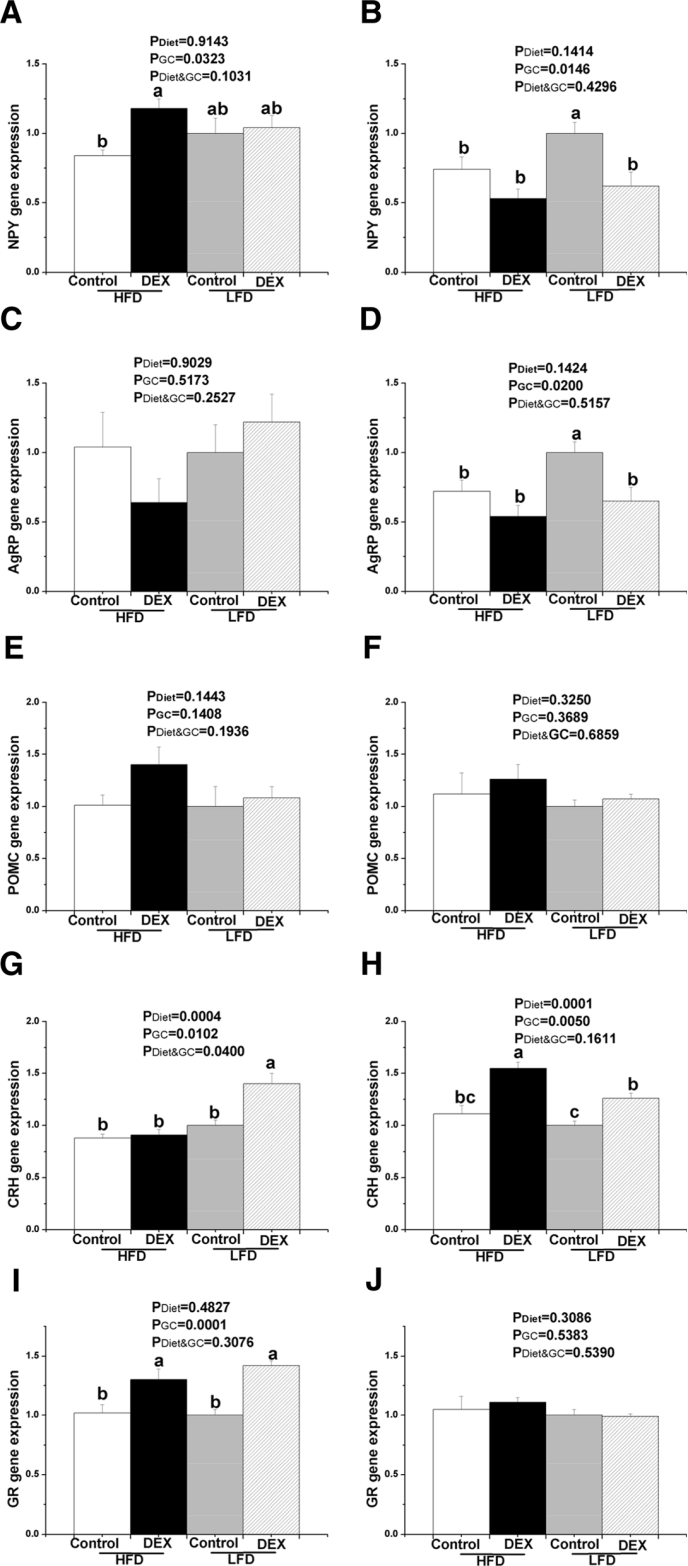
Effect of GCs on hypothalamic appetitive genes expression in chickens. **a**, **c**, **e**, **g** and **i** chickens under fasting for 24 h condition, **b**, **d**, **f**, **h** and **j** chickens under re-feeding for 3 h after fasting condition. The group with only LFD treatment was deemed to be 1. Values indicate mean ± SEM (*n* = 8). a, b Means with different superscripts are significantly different (*P* < 0.05)

Under fasting condition, DEX treatment significantly increased hypothalamic NPY and GR mRNA levels (*P* < 0.05 Fig. 3a and i). Under re-feeding condition, DEX treatment significantly decreased genes expression of NPY and AgRP (*P* < 0.05, Fig. 3b and d). No effect of dietary energy level or the interaction between DEX injection and dietary fat level was found on the hypothalamic gene expression of NPY, AgRP, POMC or GR in fasted and re-fed chickens (*P* > 0.05, Fig. 3). The chickens, given a DEX injection and a LFD, had the highest CRH mRNA levels than any of the fasted chickens given treatments (*P* < 0.05, Fig. 3g). Under re-feeding condition, a significant increase in hypothalamic CRH gene expression was found in HFD-fed chickens compared to the LFD-fed chickens (*P* < 0.05, Fig. 3h). And DEX treatment significantly increased the CRH gene expression compared to control (*P* < 0.05, Fig. 3h). An interaction effect of DEX injection and dietary fat was observed in the CRH gene expression in fasted chickens (*P* < 0.05, Fig. 3g), but not in re-fed chickens (*P* > 0.05, Fig. 3h).

## Discussion

In the present study, we investigated the effect of peripheral GCs on appetitive peptides of chickens in different energy situations. Our data show the following: 1) peripheral GCs increased hypothalamic NPY level in fasted chicks and CRH levels in re-fed chickens but decreased NPY and AgRP levels in re-fed chickens; 2) An interaction effect of DEX injection and dietary fat was observed in the CRH gene expression in fasted chickens. The chickens, given a DEX injection and a LFD, had the highest CRH mRNA levels than any of the fasted chickens given treatments. The results suggest that a regulatory network formed by NPY, AgRP and CRH is associated with the appetite-control of GCs.

### Diet rich in energy and fat alters the glucose/insulin response to GCs

GCs have an extensive effect in maintaining energy homeostasis. In line with previous findings [24], DEX significantly increased plasma glucose level regardless of feeding state. The result indicated that DEX treatment resulted in hyperglycaemia in both LFD and HFD chickens. The increased insulin level at re-fed state by DEX suggests the insulin resistance in DEX-chickens, in accordance with a previous publication [24]. At re-fed state, however, LFD-chicken had higher glucose and insulin levels compared with HFD-chickens, suggesting that the chickens fed with LFD are more prone to develop insulin resistance by DEX challenge, compared to the chickens fed with HFD. In line with this result, our previous study showed that chickens fed with LFD could have a more severe glucose response to long-term (7 days) corticosterone exposure [25]. Collectively, the result suggests that dietary energy level could alter the glucose response to GCs challenge.

### Diet rich in energy and fat alters the gene expression of orexigenic peptides

In consistent with the findings in mammals [26], body weight increased and food intake decreased significantly in HFD group compared with LFD group in this study. In crassicaudata, isocaloric diet with 10, 20, or 40 % of calories from fat resulted in altered food consumption [27]. The result indicated that chickens could adjust their food intake according to dietary energy level.

Xu et al. [28] found that the appetite alteration by HFD is related to the hypothalamic neuropeptides. In the present study, the effect of energy state on gene expression of orexigenic peptides in hypothalamus was further investigated in chickens challenged with GCs. Chicks receiving ICV NPY treatment exhibited marked hyperphagia [29]. ICV GCs injection induced high NPY gene expression in chicks [9, 30]. At fasting state, the increased hypothalamic NPY mRNA level by GCs treatment was only detected in HFD-chickens, suggesting that the effect of peripheral GCs injection on NPY expression is dependent on dietary energy concentration. In line with the results, our previous report in laying hens feeding with normal diet showed that GCs treatment had no effect on the NPY mRNA level [31]. Furthermore, the decreased NPY mRNA level by DEX was observed in LFD-chicken at re-fed state. The result implied that the regulation of GC on NPY expression is dependent on dietary energy level and feeding state.

AgRP is another orexigenic peptide in mammals and poultry [32, 33]. The co-expression of AgRP and NPY mRNA has also been observed in the infundibular nucleus of avian species [34]. In controversy to our previous study in laying hens [31], GCs treatment didn’t affect AgRP level in fasted chickens. However, there is a distinct expression of genes related to energy homeostasis and obesity in layer and broiler chickens [22]. At re-fed state, both AgRP and NPY genes expression were down-regulated by GCs treatment in LFD-chickens rather than in HFD-chickens, indicating that the regulation of GCs on orexigenic gene expression dependent on dietary energy level. Our recent study proved that GCs could evoke a special appetite on energy-rich diet rather than low-energy diet [35]. The result suggests that the regulation of GCs on orexigenic neuropeptides expression is dependent at least partially on dietary energy level and feeding state. GCs increased food consumption on the same basis of body weight [11, 12]. Hence, this result implies that peripheral GCs stimulate feed consumption by upregulating the orexigenic gene expression.

### Diet rich in energy and fat alters the feedback signalling of peripheral GCs

Hypothalamic POMC is an anorexigenic neuropeptide in both chickens and mammals [36, 37]. Fasting, ICV GC injection or peripheral GC administration have no significant effect on hypothalamic POMC mRNA level in avian species [9, 14, 31]. In accordance with the previous works, the present result further proved that peripheral GCs challenge, fasting, and re-fed had no influence on POMC gene expression.

CRH is primarily located in the periventricular part (PVH) of the hypothalamus [38]. ICV CRH injection significantly decreased feed intake in both fed and overnight- fasted birds [39]. Peripheral GCs act on the hypothalamus and pituitary to suppress CRH and adrenocorticotropic hormone production in a negative feedback cycle [40]. In this study, peripheral DEX injection increased CRH mRNA level, indicating that peripheral GCs paly a feedback effect on hypothalamic CRH gene expression. As this feedback effect was not observed in HFD-chicken at fasting state, we speculated that high-energy diet can attenuate the feedback effect of GCs on hypothalamic CRH gene expression. However, this speculation needs to be proved further.

## Conclusion

A regulatory network formed by NPY, AgRP and CRH is associated with the appetite-control by GCs. The result suggests that the regulation of GCs on orexigenic neuropeptides expression is dependent at least partially on dietary energy level and feeding state.

## Additional file

Additional file 1: Table S1. The composition and nutrient levels of the experimental diets for chickens (air dry basis). (DOC 42 kb)

## Abbreviations

NPY: neuropeptide Y
AgRP: agouti-related peptide
POMC: proopiomelanocortin
CRH: corticotropin-releasing hormone
ARC: arcuate nucleus
GCs: glucocorticoids
DEX: dexamethasone
ICV: intracerebroventricular
GR: GC receptors
INSR: insulin receptors
HFD: feeding with high-fat diet
LFD: feeding with low-fat diet
GAPDH: glyceraldehyde 3-phosphate dehydrogenase
18SrRNA: 18S ribosomal RNA
