# Supplementary material for: Effect of dexamethasone on hypothalamic expression of appetite-related genes in chickens under different diet and feeding conditions
Source: J Anim Sci Biotechnol. 2016 Apr 12;7:23. doi: 10.1186/s40104-016-0084-x (PMC4828879; doi:10.1186/s40104-016-0084-x)
Supplement: Additional file 1: Table S1. — The composition and nutrient levels of the experimental diets for chickens (air dry basis). (DOC 42 kb) [file 40104_2016_84_MOESM1_ESM.doc]

**Table 1.** The composition and nutrient levels of the experimental diets for chickens (air dry basis)

| Ingredients (%) | LFD | HFD |  | Calculated chemical composition | LFD | HFD |
| --- | --- | --- | --- | --- | --- | --- |
| Maize | 46.622 | 44.859 |  | Metabolism energy (MJ/Kg) | 10.900 | 15.060 |
| Soybean meal | 30.676 | 36.984 |  | Crude protein % | 20.000 | 20.000 |
| Choline chloride | 0.260 | 0.260 |  | Met % | 0.531 | 0.556 |
| Dicalcium phosphate | 1.424 | 1.643 |  | Lys % | 0.964 | 1.073 |
| Mountain flour | 1.893 | 1.742 |  | Ca % | 1.000 | 1.000 |
| Methionine | 0.235 | 0.294 |  | P % | 0.450 | 0.450 |
| Lysine | 0.171 | 0.246 |  |  |  |  |
| NaCl | 0.255 | 0.255 |  |  |  |  |
| Bean oil | 0 | 13.468 |  |  |  |  |
| Bran | 18.214 | 0 |  |  |  |  |
| Premix^*^ | 0.250 | 0.250 |  |  |  |  |

*Premix provides the following per kg of diets: VA, 8 000 IU; VD3, 3 000 IU; VE, 20 IU; VK, 2 mg; VB1, 4 mg; riboflavin, 8 mg; D-pantothenic acid, 11 mg; VB5, 40 mg; VB6, 4 mg; VB12, 0.02 mg; biotin, 0.15 mg; folic acid, 1.0 mg; choline, 700 mg; Fe (as ferrous sulfate), 80 mg; Zn (as zinc sulfate), 75 mg; Mn (as manganese sulfate), 80 mg; Cu (as copper sulfate) 10 mg, I (as potassium iodide), 0.40 mg; and Se (as sodium selenite), 0.30 mg.
